# Fifty years of data show the effects of climate on overall skull size and the extent of seasonal reversible skull size changes (Dehnel's phenomenon) in the common shrew

**DOI:** 10.1002/ece3.9447

**Published:** 2022-10-27

**Authors:** Jan R. E. Taylor, Marion Muturi, Javier Lázaro, Karol Zub, Dina K. N. Dechmann

**Affiliations:** ^1^ Faculty of Biology University of Białystok Białystok Poland; ^2^ Department of Migration Max Planck Institute of Animal Behavior Radolfzell Germany; ^3^ Department of Biology University of Konstanz Konstanz Germany; ^4^ Mammal Research Institute Polish Academy of Sciences Białowieża Poland

**Keywords:** body size, climate change, Dehnel's phenomenon, shrews, skull size

## Abstract

Global climate change affects many aspects of biology and has been shown to cause body size changes in animals. However, suitable datasets allowing the analysis of long‐term relationships between body size, climate, and its effects are rare. The size of the skull is often used as a proxy for overall body size. Skull size does not change much in fully grown vertebrates; however, some high‐metabolic small mammals shrink in winter and regrow in spring, including their skull and brain. This is thought to be a winter adaptation, as a smaller brain size reduces energy requirements. Climate could thus affect not only the overall size but also the pattern of the size change, that is, Dehnel's phenomenon, in these animals. We assessed the impact of the changes in climate on the overall skull size and the different stages of Dehnel's phenomenon in skulls of the common shrew, *Sorex araneus*, collected over 50 years in the Białowieża Forest, E Poland. Overall skull size decreased, along with increasing temperatures and decreasing soil moisture, which affected the availability of the shrews' main food source, earthworms. The skulls of males were larger than those of females, but the degree of the decrease in size did not differ between sexes. The magnitude of Dehnel's phenomenon increased over time, indicating an increasing selection pressure on animals in winter. Overall, climate clearly affected the common shrew's overall size as well as its seasonal size changes. With the current acceleration in climate change, the effects on the populations of this cold‐adapted species may be quite severe in a large part of its distribution range.

## Graphical abstract

We assesed the impact of the changes in climate on the overall skull size (the proxy of the overall body size) and the seasonal changes of skull height (Dehnel's phenomenon) in skulls of the common shrew, *Sorex araneus*, collected over 50 years in the Białowieża Forest, NE Poland. Overall skull size decreased, along with increasing temperatures and decreasing soil moisture, which determined the availability of the shrews' main food source, earthworms. The magnitude of Dehnel's phenomenon increased over time, indicating an increasing selection pressure on animals in winter.

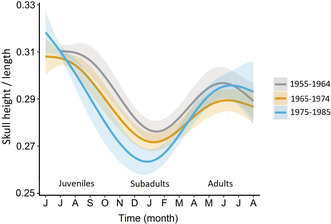

## 1 INTRODUCTION

Global climate change affects animals in many ways, from phenology, geographic distribution, and phenotypic traits to distributions and population dynamics (Gardner et al., 2011; Parmesan, 2006; Walther et al., 2002). Changes in phenotypic traits correlated with global warming mainly involve temporal trends in body mass and size (Gardner et al., 2011), two traits that are pivotal for individual life histories (Sauer & Slade, 1987). Importantly, although mass and size are often used interchangeably, their change arises from different underlying physiological mechanisms. Mass is more sensitive to short‐term environmental conditions affecting fat stores and the mass of the gastrointestinal tract and other organs (Canale et al., 2016; Hume et al., 2002; Piersma et al., 1999).

How climate influences body size is often described based on Bergmann's rule, which in its commonly used version describes a positive relationship between body size and latitude of occurrence within species. Individuals of many homeothermic species are smaller at lower latitudes where temperatures are higher (e.g., Ashton, 2002; Ashton et al., 2000). This implies that a similar relationship should occur in response to temperature change over time. It has been postulated that a decrease in body size is the third universal response to contemporary global warming in addition to changes in distribution and phenology (Gardner et al., 2011). Although the majority of studies did not find significant temporal changes in the sizes of birds and mammals, there are many examples of long‐term body size and body mass changes (recorded over one to several decades) correlated with global climate change. While a decrease in size is indeed the major response in birds, the situation is much less clear in mammals, where many species increase in size (Naya et al., 2017; Nengovhela et al., 2020; Teplitsky & Millien, 2014). This increase in size is usually explained as a reaction to increased food availability as a result of increasing temperatures (Boutin & Lane, 2014 and references cited therein; Yom‐Tov & Geffen, 2011).

Soricine (red‐toothed) shrews, especially *Sorex* shrews, are an excellent study system to investigate climate change‐induced patterns in species‐level body size changes. They have extremely high metabolic rates, much higher than expected for their body mass (Ochocińska & Taylor, 2005; Taylor, 1998). Consequently, shrews require a constant high food supply (Hanski, 1994; Keicher et al., 2017). In addition, the body size of several *Sorex* species, represented by skull length, shows positive correlations with temperature and negative correlations with latitude across their distribution range, contrary to Bergmann's rule (Ochocińska & Taylor, 2003). It was suggested that food scarcity during winter in cold northern climates is a major factor selecting for small body size in shrews. This is in line with the “Resource Rule” of McNab (2010), which posits that “mammalian species will become larger or smaller depending on the size, abundance and availability of resources.” A decrease in body size with latitude and in cold areas was also found in the masked shrew, *Sorex cinereus*, in Alaska and interpreted as related to better food resources in warmer areas (Yom‐Tov & Yom‐Tov, 2005). In agreement with this, the body size of *S. cinereus* in Alaska increased during the second half of the twentieth century along with increasing temperature, presumably due to increasing food availability in winter (Yom‐Tov & Yom‐Tov, 2005). However, an increasing temperature, especially when coupled with a decrease in precipitation, can also lead to a decrease in food resources. Drought has a negative impact on the abundance of shrews invertebrate prey (Coyle et al., 2017). This includes earthworms (Lumbricidae), the main food of the common shrew, *Sorex araneus* (Shchipanov et al., 2019), the availability of which depends on high soil moisture (Coyle et al., 2017; Singh et al., 2019).

The unfavorable seasonal food conditions (caused directly or indirectly by climate) may bring about the advantage of being smaller in seasons when food is scarce. This is visible in the remarkable seasonal changes in size and morphology exhibited by soricine shrews. The size of their skull and brain undergo a profound seasonal and reversible transformation: seasonal shrinkage and regrowth of individuals, whose magnitude is only comparable with that in some mustelid species (Dechmann et al., 2017; LaPoint et al., 2017; Lazáro et al., 2017). Skull (braincase) height, but not skull length, decreases in winter, resulting in a flattened skull shape. This change in the skull, brain, and many other organs and tissues, as well as the body mass of soricine shrews and some other small high‐metabolic animals, is called Dehnel's phenomenon (Dechmann et al., 2017; Dehnel, 1949; Pucek, 1963, 1970). After juveniles are fully grown, their brain mass decreases by 21% on average, and skull height decreases by 13% in *S. araneus*. Their brain mass reaches a minimum value in winter and then partially regrows in spring (Lazáro et al., 2021). Body mass, in contrast, decreases in anticipation of winter but then almost doubles in spring. Spring growth is related to reaching sexual maturity and likely to improved weather and food conditions. *Sorex araneus*, born in early summer, has a maximum lifespan of approximately 14 months, and almost all individuals die before the second winter after a terminal reproductive period (Pucek, 1981). The size decrease in winter is thought to be an adaptation to harsh climatic conditions in these nonhibernating, high‐metabolic animals. The low body mass in shrews in winter has been hypothesized to reduce absolute food requirements when food availability is limited (McNab, 1991; Mezhzherin, 1964; Taylor et al., 2013). Accordingly, small winter animals have the same mass‐corrected energy consumption as larger, first‐ and second‐summer shrews, even under ambient conditions with temperatures differing by as much as 30°C (Schaeffer et al., 2020). This results in absolute energy savings in winter and correlates with reduced food requirements. Reducing the size of the brain, an energetically expensive tissue (Aiello & Wheeler, 1995; Isler & van Schaik, 2006), may lead to further energy savings. Additionally, the decline in the relative weight of the liver, spleen, and adrenals and decreased synthesis by the endocrine glands in winter may contribute to lower metabolic rates in *S. araneus* and compensate for the increase in relative heart mass during this time (Hyvärinen, 1984; Pucek, 1965).

The magnitude of the winter decline in skull height in *S. araneus*, a characteristic component of Dehnel's phenomenon, increases toward the northeast in Europe and is linked to large‐scale environmental conditions and probably also the local habitat structure (Lazáro et al., 2021; Pucek, 1970). It is positively correlated with temperature seasonality, annual temperature range, and other climate parameters, although no such relationships have been observed with skull height regrowth (Lazáro et al., 2021), which has led to the hypothesis that shrinking may be the result of different evolutionary drivers than regrowth. In line with these observations, the individual decrease in skull height in *S. araneus* is flexible and modulated by ambient temperature (Lazáro et al., 2019).

Our aim was to reject a null hypothesis that the change in climate over several decades has had no impact on (1) the overall size of *S. araneus* as measured by skull dimensions and (2) the intensity of the reversible seasonal size change (Dehnel's phenomenon). We combined climate data and skull size measurements of *S. araneus* from a 52‐year series of specimens collected in the Białowieża Primeval Forest, E Poland, from 1953 to 2004.

We first hypothesized that H1: because they are larger at lower, warmer latitudes, the body size of *S. araneus* increased over the years as a response to increasing temperatures (and increasing food availability as in the case of *S. cinereus*) or Ha1: decreased as a response to a drier climate leading to a decreasing soil water level and thus lower earthworm availability in the Białowieża Forest. We further hypothesized that H2: increasing winter temperatures reduce the winter decrease in skull height (and thus brain size). Conversely, Ha2: the winter decrease in skull height will become greater if climate change is combined with increasingly less favorable food availability in winter than in summer.

## 2 METHODS

### 2.1 Shrews, study area and trapping

**FIGURE 1:**
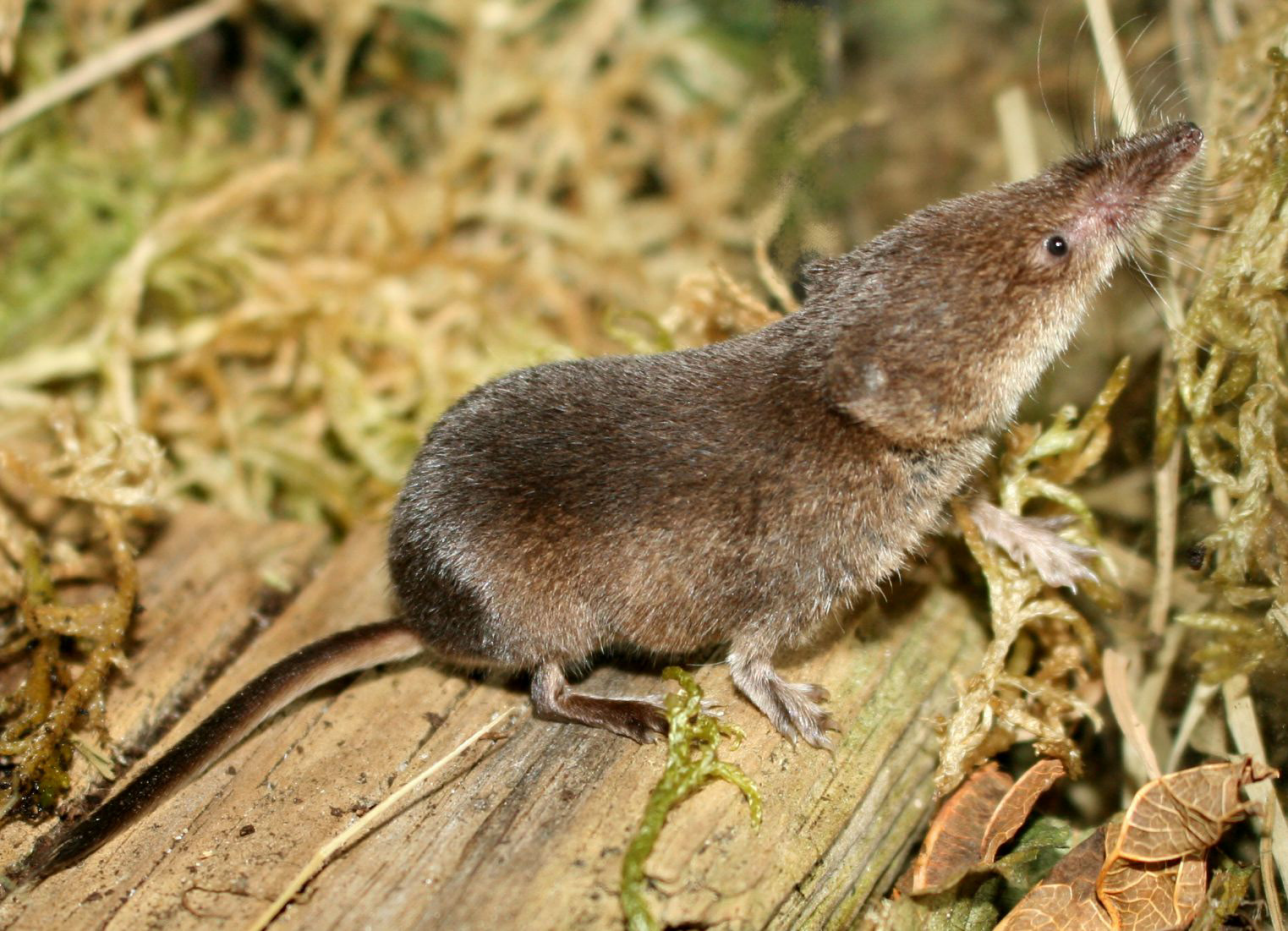
The common shrew (*Sorex araneus*). Photo by Leszek Rychlik.

*Sorex araneus* (Figure 1) were collected between 1953 and 2004 in Białowieża National Park, preserved in alcohol and stored in the zoological collection of the Mammal Research Institute, Polish Academy of Sciences, in Białowieża. Shrews were trapped in a mesic broadleaved forest with a transition to a moist broadleaved forest, mainly in compartment No. 371 (52°44′N, 23°52′E) but also in adjacent Nos. 369 and 370 (see Dehnel, 1949 for a map; all these compartments are in the strict reserve). This type of forest is classified as a subcontinental oak‐lime‐hornbeam forest *Tilio Carpinetum*. The mean age of the trees exceeded 100 years, and the amount of dead wood on the ground averaged 50 m^3^ ha^−1^.

*Sorex araneus* were caught with pitfall traps (cylinders 30 cm deep and cones 45 cm deep). After 1965, wooden box live traps were additionally used. We selected and measured the skulls of shrews from the three main stages of Dehnel's phenomenon: sexually immature summer individuals in their first calendar year (hereafter “juveniles”) caught from July to August; smaller, immature, winter individuals (“subadults”) caught from December to February; and regrown, sexually mature spring/summer individuals in their second calendar year (“adults”) caught from April to August. The individuals were assigned to the above three age classes upon capture, and their age was recorded in the collection database. Their sex was determined by dissection. Juveniles are easily distinguishable from adults by the degree of tooth wear, development of gonads, and fur appearance (Churchfield, 1990; Pankakoski, 1989). Because *S. araneus* lives for approximately 1 year and only reproduces in spring and summer, there is no generation overlap of mature adults. In winter, there is only one age class—subadults. After 1981, juvenile individuals were trapped only in July. There was no winter trapping at all after 1985. We also measured three individuals each from June (juveniles) and November (subadults) and two from September (juveniles) from 1955 to 1985.

### 2.2 Measurements of skull dimensions

**FIGURE 2:**
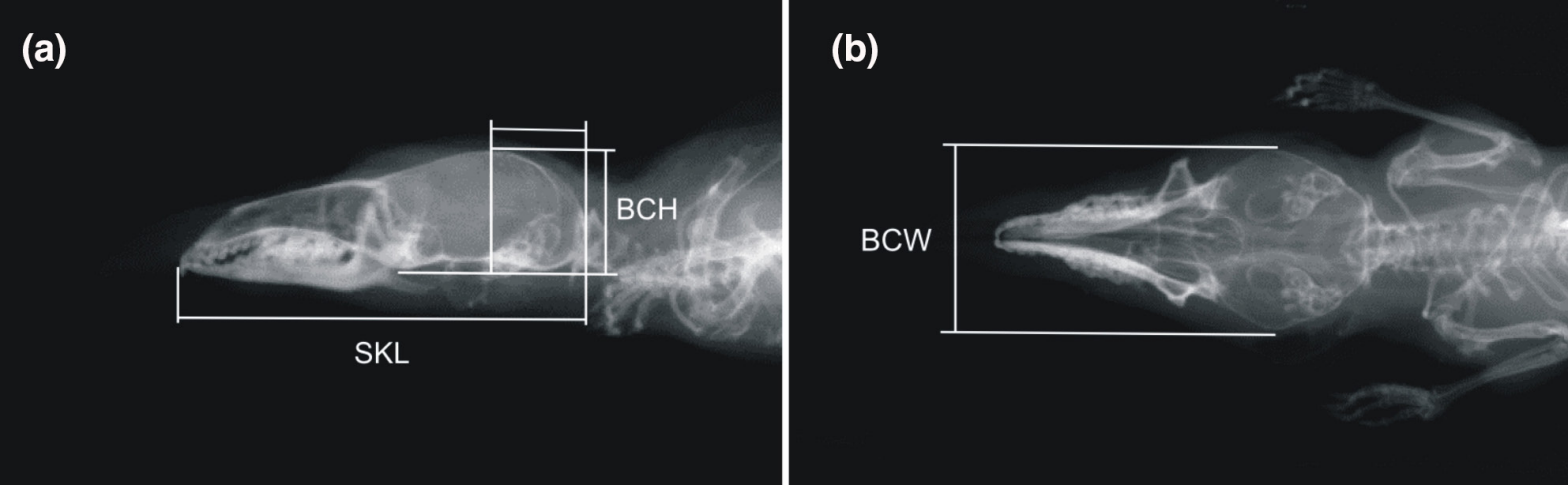
X‐ray images of *Sorex araneus* skulls and linear measurements taken from the images: (a) skull length (SKL), skull (braincase) height (BCH), and (b) skull width (BCW). See text for details.

A single observer measured the skull height, length, and width (mm) using X‐ray images of the alcohol‐preserved specimens, as described by Lazáro et al. (2017). Briefly, skull length was measured from the anterior‐most projection of the first incisive tooth to the occipital condyle (Figure 2a); skull height was measured from the dorsal outline of the braincase to the orthogonal line defined by the ventral outlines of the pterygoid process and occipital condyle, passing over a constant proportional distance on the line defined by the skull length (Figure 2a); skull width was the greatest lateral diameter of the braincase (Figure 2b). The technical error of measurement (standard deviation of the repeated measurements of the same individual as the % of the mean) for the skull length, height, and width was 1.35%, 2.04%, and 1.89%, respectively (supplemental information in Lazáro et al., 2017). We measured a total of 502 skulls.

### 2.3 The choice of months representing the three age categories

**FIGURE 3:**
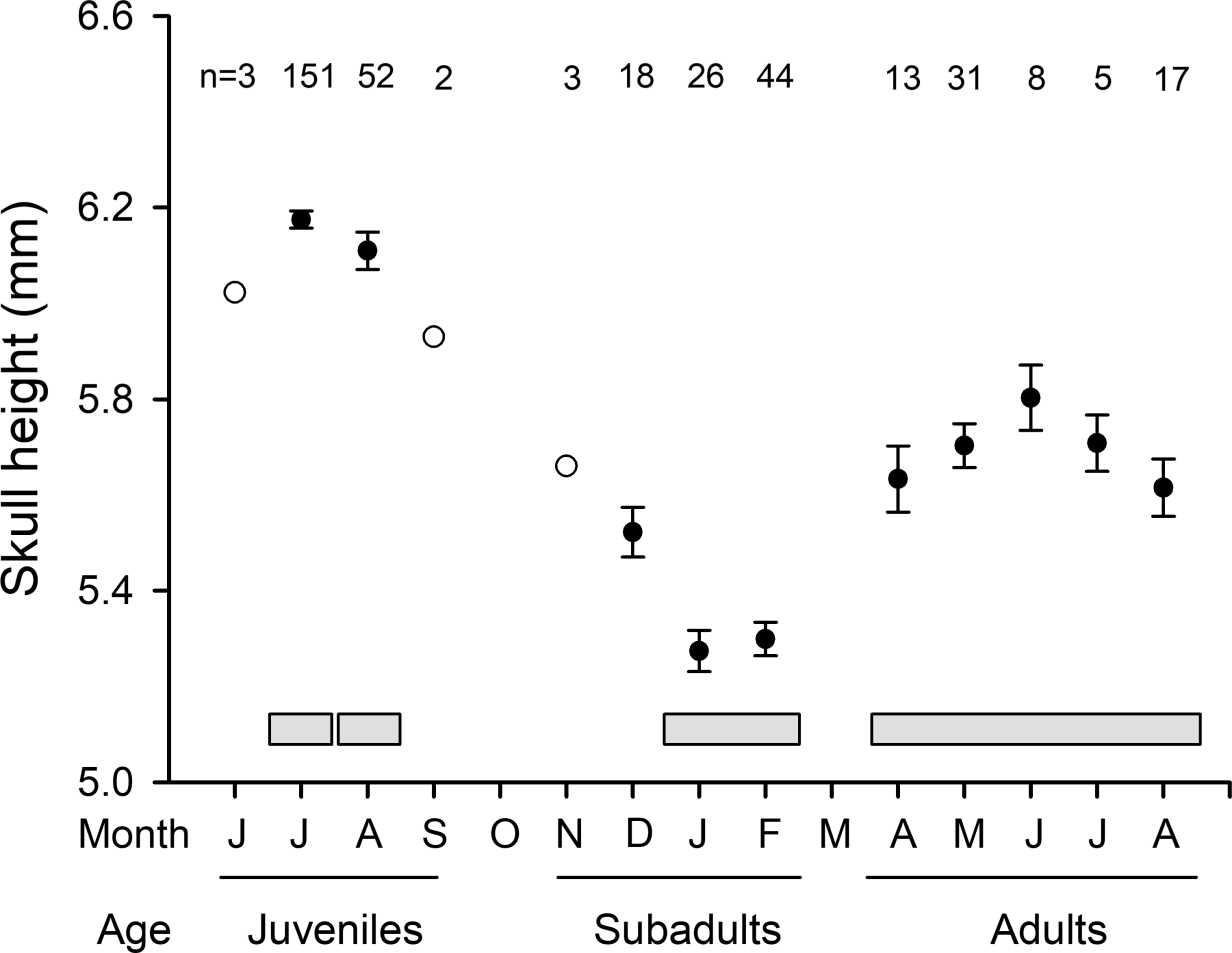
Skull height throughout the lifetime of *Sorex araneus* from Białowieża Forest. Sampling months and corresponding age categories are shown. The first weaned shrews appeared in June (juveniles); they then shrank toward winter (subadults) and finally regrew in the next spring/summer (adults), when the adults reproduced. Most of the shrews then died before the next winter. Solid circles are the means ± standard errors when the sample size was ≥5, open circles are the means with sample size <5. Horizontal bars represent age categories and months from which skulls were analyzed; skulls of juvenile shrews from July and August were analyzed separately (see text). The presented data were limited to the period between 1955 and 1985, for which we had individuals from all three age categories.

We compared skulls in three age categories of individuals who characterize their entire lifetime: fully grown juveniles, size‐decreased subadults, and regrown adults (Figure 3). To test the homogeneity of each age category, we used an ANCOVA model with the month as a factor, the time (year) as a covariate, and the interaction between month and year. The skull height of juvenile individuals differed between July and August (*F*_1,201_ = 8.08, *p* = .0049), and the interaction was significant (*F*_1,201_ = 8.12, *p* = .0048) in the period between 1953 and 1981, when individuals from both months were measured. Thus, we further analyzed July and August juveniles separately.

We chose the measurements of the skull heights from January to February, when they are the lowest during the lifespan of *S. araneus* (Figure 3), as a representative for the subadult age category. The interaction between year and month, as well as month, was nonsignificant (*F*_1,66_ = 0.04, *p* = .839; *F*_1,66_ = 0.04, *p* = .836). The difference between the 2 months was also nonsignificant (*F*_1,67_ = 0.55, *p* = .461) when the interaction was removed from the model.

In adults (Figure 3), neither the year × month interaction nor month was significant in the model for skull height (*F*_4,104_ = 0.88, *p* = .476; *F*_4,104_ = 0.88, *p* = .477). Month was also not significant (*F*_4,108_ = 1.67, *p* = .163) when the interaction was removed from the model. Thus, we retained all months from April to August in the final analyses of adults.

**FIGURE 4:**
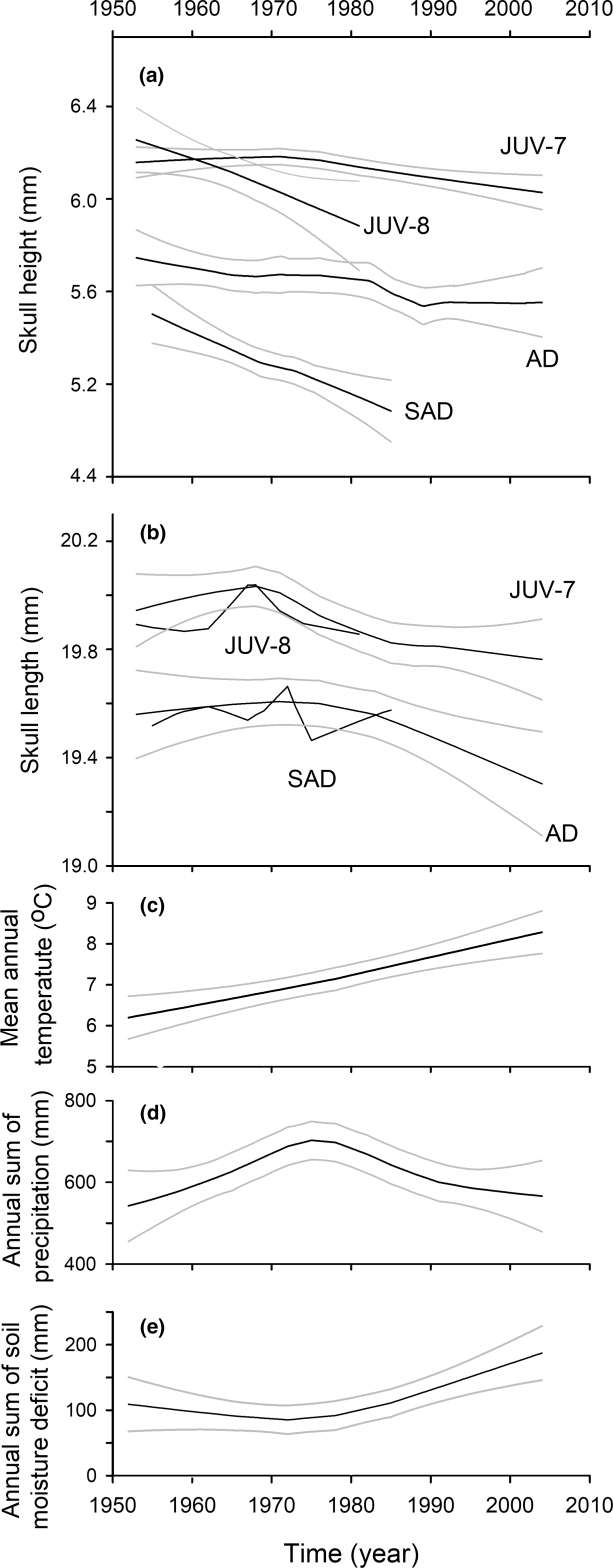
The relationships between (a) skull height, (b) skull length and year in July juveniles (JUV‐7; 1953–2004), August juveniles (JUV‐8; 1953–1981), subadults (SAD; 1955–1985) and adults (AD; 1953–2004) of *Sorex araneus* compared with changing climate parameters: (c) the mean annual temperature, (d) the annual sum of precipitation, and (e) the annual sum of soil moisture deficit. The 95% confidence limits of the relationships smoothed with LOESS are shown (for the sake of clarity, confidence limits for JUV‐8 and SAD in (b) are not presented). Additionally, for clarity, both sexes are combined in (a) and (b) (the difference in skull size between sexes was small and the change with time was parallel; see text).

We ran the same tests for skull width and length, which vary much less between seasons (Lazáro et al., 2017). The year × month interaction was not significant in any age category, and there were no significant differences between collection months. Including sex in the above models did not change the results. Table 1 shows the numbers of animals/skulls used in the analyzed age categories. See also Figure 4 for an overview of the analyzed *S. araneus* data.

**TABLE 1:** Numbers of *Sorex araneus* males and females in the analyzed age categories

Period	Age	Numbers		
		Males	Females	Total
1953–2004	Juveniles (July)	126	108	234
	Adults (April–August)	88	26	114
1955–1985	Juveniles (July)	86	65	151
	Juveniles (August)	45	7	52
	Subadults (January–February)	43	27	70
	Adults (April–August)	64	10	74

*Note*: Only July juveniles and adults were available for the whole period of 1953–2004.

The choice of months representing the three age categories rests on the assumption that the phenology of *S. araneus* did not change throughout the study period. The first juveniles of *S. araneus* appear in the Białowieża Forest at the turn of May and June. They were regularly caught in May and June and deposited in the collection until 1975. The mean date of the first capture between 1953 and 1970 was June 2 (within 11 years, when shrews were abundant; SD = 3.5 days). From 1972 to 1975, when the climate began to change (see Section 3), the dates of the first appearance did not notably change and were June 1 in 1972 to 1974 and May 27 in 1975. In 1989 and 1990, when capture occurred in both in May and July, the first juveniles were caught on May 31 and July 6. The regression of the dates of the first appearance (May 27 = 1) on time (years) was nonsignificant between 1953 and 1990 (*F*_1,15_ = 0.952, *p* = .952). In 1978, 1979, 1983, and 1987, when shrews were caught in the second half of May, but not in June, no juvenile *S. araneus* were caught, only adults were caught. In 1981, when no catching was performed in May, the first juvenile was caught on June 1. In summary, we found no signs of change in the breeding phenology, at least between 1953 and 1990. Moreover, reaching the minimum skull height in winter did not change over the years; it occurred in January and February, regardless of the decade or locality (Dehnel, 1949; Lazaro et al., 2019; Pucek, 1970).

### 2.4 The change in skull size from 1953 to 2004

We first analyzed the changes in the overall size of *S. araneus* using the full dataset from 1953 to 2004, which was only available for juveniles from July and adults. We tested the temporal change in all three skull dimensions, skull height, width, and length, with three ANCOVA models with age and sex as factors and time (year) as a covariate. We added a quadratic term to the linear models to test for linearity of the relationships and determine whether it improved the model according to an *F* test with a type I sum of squares.

We also tested the linearity of the regressions of the three dimensions over time for two age categories separately. The quadratic term was only significant for the skull length of adults (*p* = .032). However, inspection of the residuals from other linear regressions suggested a more subtle nonlinearity of the skull size over time. Thus, we additionally used locally weighed regression smoothing (LOESS; Cleveland, 1979). We selected the smoothing parameter by minimizing Akaike's information criterion, which strikes a balance between the residual sum of squares and the complexity of the fit.

### 2.5 The influence of climate change on the size of *S. araneus*

We examined the weather data from Białowieża from 1952 to 2004 to explain changes in skull size in juvenile and adult *S. araneus*. We obtained the mean daily temperature (°C), rainfall (mm), and snow cover (mm) from the Białowieża Meteorological Station. We summed or averaged these daily measures for longer periods and computed the soil moisture deficit from monthly temperatures and precipitation using the Watbug program (Willmott, 1977). Monthly deficits were summed for years, or other periods as indicated below. We used these deficits as the indicators of earthworm abundance in the soil. As in the previous section, we used linear regression, tested for nonlinearity by adding quadratic terms, and smoothed the relationships with LOESS.

We calculated the mean daily temperature, sum of the precipitation, and sum of the moisture deficit in the soil in the period preceding the collection of specimens (from their approximate time of birth; see Section 3 for details). We also used the mean temperature of January and February, the number of days with snow cover in these months and during the whole winter preceding the collection of adult *S. araneus*.

To select models best characterizing the relationship between climatic variables and skull dimensions we ordered all possible models (separately for juveniles and adults) according to the AICc (sample size corrected AIC). Then we inspected all models with delta <3 for most influential variables. Next instead of averaging coefficients for equally supported models (as for all skull measurements within the two age classes there was no single best model), we selected climatic variables which are not correlated and significantly explain most of variation (based on *R* ^2^). We checked for a temporal autocorrelation (correlation of residuals in neighboring years) for all three skull measurements using autocorrelation function (ACF) in R for a wide range of lags. Autocorrelation was not significant in any of the models.

### 2.6 Skull dimensions in all three age categories from 1955 to 1985

We compared absolute values of the skull height, width, and length for the years when specimens for all three age categories were available in an ANCOVA with age and sex as the factors and sampling year as the covariate.

### 2.7 Change in the magnitude of Dehnel's phenomenon from 1955 to 1985

We compared the amount of change in skull height from the subset where we had data from all three size extremes (summer juveniles, winter subadults, and regrown adults). To do this, we compared juveniles (July and August separately) with subadults and subadults with adults in ANCOVA models with age as the factor, year as the covariate, and the interaction between year and age.

To visualize the change in the magnitude of Dehnel's phenomenon over time, we divided the years where we had data from all three size extremes (summer juveniles, winter subadults, and regrown adults) into three time intervals (1955–1964, 1965–1974, and 1975–1985). We divided skull height by skull length (which does not change within individuals) to control for differences in overall body size between individuals. Next, we compared the seasonal curves of the relative skull height between these three time intervals with a generalized additive model (GAM) using “time interval” as a parametric term and “month” as a nonparametric term. We used a Gaussian distribution and applied a smoothing function to “month” with knots restricted to five (because we had an a priori expectation for the presence of the shape of a seasonal skull height pattern).

### 2.8 Statistics

We used general linear models (GLMs) for ANOVA and ANCOVA models. Multiple comparisons between means after the ANOVAs were performed with Tukey–Kramer tests. We tested the significance of all possible interactions between covariates and factors in all ANCOVA models and reported all significant results. The tests reported in this study were performed with procedures GLM, REG, and LOESS in SAS (ver. 9.4; SAS Institute). GAM models were fitted with the “mgcv” package in R (ver. 3.3.1; R Core Team, 2016). We used packages “lmtest” and “MuMIn” in R to fit linear models analyzing the effect of climate change on size of *S. araneus*. Three lowest, outlying measurements of the skull length of juveniles were not used in statistical analyses.

## 3 RESULTS

### 3.1 The change in skull size from 1953 to 2004

All three measures of skull size, skull height, width, and length, of juvenile and adult *S. araneus* decreased significantly over the sampling period, as revealed by ANCOVAs with age, sex and year (Table 2a, Figure 4a,b; see Figure S1 for scatterplots and regression lines). Skull height and length, but not skull width, differed between the two age categories and were larger in juveniles than in adults (Table 2a,b). The interactions between sex and time (years) were not significant when included in the models (*F*_1,343_ = 0.55, *p* = .458, skull height; *F*_1,343_ = 0.37, *p* = .543, width; *F*_1,340_ = 0.28, *p* = .598, length), which indicated that male and female skulls were shrinking at the same rate. All three measures were larger in males than in females, except for adult skull length (Table 2a,b, Figure S1). The largest difference between the two sexes was in skull height: 2.8% in adults and 1.1% in juveniles (Table 2b). Characteristically, all relationships of skull dimensions with time (except skull height in adults) revealed a common pattern when smoothed with LOESS: little change until the early 1970s and decreases thereafter (Figure 4 and Figure S2).

**TABLE 2:** (a) Results of three ANCOVA models testing the relationship between skull height, width, and length and age category (juveniles from July and adults), sex, and year of sampling in *Sorex araneus* in Białowieża Forest from 1953 to 2004; *n* = 348 individuals (*n* = 345 in skull length; without three outliers in juveniles). All significant effects are marked in bold. All four possible interactions added to each of three models were nonsignificant (b) Mean dimensions of skulls (mm) with standard errors for juvenile and adult males and females; different letters (a, b) mark significant differences of a given dimension between the sexes in the same age category.

	Skull		
	Height	Width^†^	Length
**(a)**			
Age	*F*_1,344_ = 426.9 *p* < **.0001**	*F*_1,344_ = 0.03 *p* = .867	*F*_1,341_ = 101.7 *p* < **.0001**
Sex	*F*_1,344_ = 11.2 *p* = **.0009**	*F*_1,344_ = 11.7 *p* = **.0007**	*F*_1,341_ = 10.58 *p* = **.0013**
Year	*F*_1,344_ = 13.3 *p* = **.0003**	*F*_1,344_ = 13.1 *p* = **.0003**	*F*_1,341_ = 12.7 *p* = **.0004**
*R* ^2^	.565	.079	.258
**(b)**			
Juvenile males	6.17 ± 0.02^a^	10.19 ± 0.03^a^	19.98 ± 0.03^a^
Juvenile females	6.10 ± 0.02^b^	10.11 ± 0.02^b^	19.83 ± 0.04^b^
Adult males	5.67 ± 0.03^a^	10.24 ± 0.03^a^	19.58 ± 0.04^a^
Adult females	5.51 ± 0.04^b^	10.00 ± 0.06^b^	19.47 ± 0.07^a^

^†^ The quadratic term was significant when added to the model (*F*_1,343_ = 4.09, *p* = .044); age: *F*_1,343_ = 0.02, *p* = .876; sex: *F*_1,343_ = 11.28, *p* = .0009; year *F*_1,343_ = 4.04, *p* = .045; *R* ^2^ = .089. The interaction between sex and year when added: *F*_1,342_ = 0.62, *p* = .432.

### 3.2 The influence of climate change on the size of *S. araneus*

The weather records from this period revealed several significant trends indicative of a changing climate (scatterplots of six climate parameters are shown in Figure S3). The mean annual temperature increased significantly (*F*_1,51_ = 28.1, *p* < .0001; Figure 4c and Figure S3a) from 6.1 to 8.2°C (as calculated from the regression line). There was also an increase in the mean July temperature (*F*_1,51_ = 10.5, *p* = .0021; Figure S3b), from 17.2 to 19.7°C. The mean January temperature decreased until 1972 (*F*_1,17_ = 26.1, *p* < .0001, calculated without two clear outliers; Figure S3c) and increased thereafter. The relationship of the sum of annual precipitation with time was nonlinear (quadratic term: *F*_1,50_ = 9.30, *p* = .0037), with an initial increase leading to a maximum in the mid‐1970s, followed by a decrease until 2004 (the decrease in 1971–2004 was statistically significant: *F*_1,32_ = 11.25, *p* = .0021, linear regression) (Figure 4d and Figure S3d). The period of high precipitation was mainly due to increased rainfall in June and July. Continuous increases in mean annual and mean July temperatures coupled with a significant decline in precipitation resulted in an increased nonlinear soil moisture deficit over the years (quadratic term: *F*_1,50_ = 7.69, *p* = .0078; Figure 4e and Figure S3e). This change in the deficit was not significant until 1971 (*F*_1,18_ = 0.20, *p* = .660, linear regression), but there was a significant increase in the soil moisture deficit after 1971 (*F*_1,32_ = 11.9, *p* = .0016). The number of days with snow cover significantly decreased over the study period (*F*_1,51_ = 4.57, *p* = .0374) (Figure S3f). Interestingly, the decrease in precipitation, the beginning of the increase in drought, and the end of the decrease in January temperature in the early and mid‐1970s corresponded with the inflection points of the smoothed relationships of the skull size versus years and with the clear decrease in skull size later on (Figure 4). To discuss the impact of weather on Dehnel's phenomenon, we additionally tested the regressions of all weather parameters against time (years) in July, August, and January between 1955 and 1985. Only the August mean daily temperature was significant (positive relationship; *F*_1,29_ = 6.36, *p* = .0175).

**FIGURE 5:**
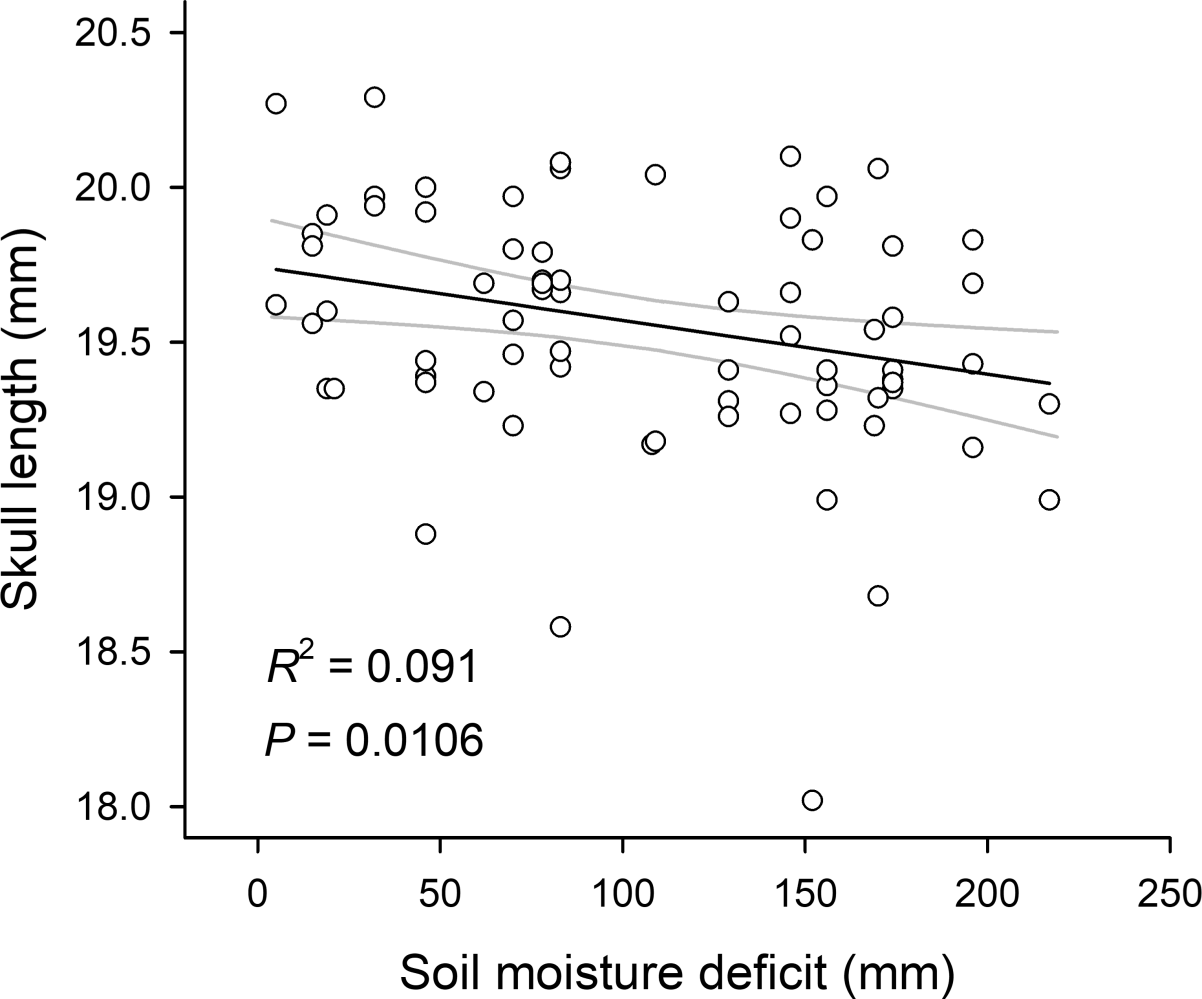
Relationship between the skull length in adult *Sorex araneus* and the soil moisture deficit; regression line with the 95% confidence limits; the lowest, outlying point of skull length was omitted from the regression. The period for which the soil moisture deficit was calculated is explained in Table 3.

We found that soil moisture deficit and precipitation were correlated with skull size in *S. araneus* and could be the drivers of skull size decrease over years (Table 3). Although skull length, the proxy of the overall body size, was not correlated with climate parameters in adults in the whole time series (between 1953 and 2004), it was significantly correlated with soil moisture deficit between 1971 and 2004 (Table 3), in the period of significant increase in drought; an increased deficit was correlated with a shorter skull length (Figure 5). The model of skull height of juveniles in the whole time series included the soil moisture deficit, but it did not reach the significance level (*p* = .070). In the whole time series, skull width of adults increased significantly with the increase in precipitation. The model of skull length of juveniles between 1971 and 2004 included the positive correlation with precipitation (*p* = .080). We also found an impact of winter weather (during the subadult stage) on adult skull height the following summer: summer skull heights were greater when there were more days with snow cover in the previous January and February (Table 3).

**TABLE 3:** The relationship of the change in skull height, length, and width in juvenile (July) and adult *Sorex araneus* with weather variables between 1953 and 2004, and in skull length between 1971 and 2004 (the period of significant increase in drought).

	Model	*R* ^2^	*p*
Juveniles 1953–2004			
Height	Sex* − Year* − SumDeficit	.072	.0007
Length	Sex*** − Year* − Temp	.111	<.0001
Width	Sex* − Year**	.057	.001
Adults 1953–2004			
Height	Sex** + SnowJF**	.164	<.0001
Length	Sex	.022	.113
Width	Sex** − Year + SumPrec*	.159	.0003
Juveniles 1971–2004			
Length	Sex** − Year + SumPrec	.115	.0006
Adults 1971–2004			
Length	Sex − SumDeficit*	.132	.0083

*Note*: SumDef, T, and SumPrec are shrew's “whole‐life” weather parameters; for juveniles, they were calculated for the period between May and June, which covers the early life of juveniles and preceded their collection in July; for adults, they were calculated from June, when the first cohort of shrews was weaned, to March of the following year, when adult shrews were collected between April and August. SnowJF = the number of days with a snow cover in January and February preceding the collection of adults. *R* ^2^ is the proportion of the variance for the skull size explained by the independent variables in the multiple regression. Significance of the independent variables: * = 0.05; ** = 0.01; *** = 0.001.

Abbreviations: SumDef, the sum of monthly soil moisture deficit; SumPrec, the sum of precipitation; T, the mean temperature.

### 3.3 Skull dimensions in all three age categories from 1955 to 1985

In the time period from 1955 to 1985, we had measurements from all three ages: juveniles, subadults, and adults. Skull height, width, and length were significantly correlated with age (confirming the typical seasonal size change pattern; Table 4a). Only skull height showed a negative significant relationship with year, and the interaction between year and age was significant when included in this model.

**TABLE 4:** (a) Results of the ANCOVA testing the relationship between skull height, width, and length and age category (juveniles from July, subadults, and adults), sex, and year of sampling in *Sorex araneus* in Białowieża Forest from 1955 to 1985. Significant effects are marked in bold. Models of width and length do not include the interaction between age and year because it was nonsignificant (b) Mean dimensions of skull (in mm) with standard errors, reported for three age categories; different letters (a, b, c) in a given column mark significant differences between age categories (at *p* = .05)

	Skull			
	*n*	Height	Width	Length^†^
**(a)**				
Age	295	*F*_2,288_ = 4.32 *p* = **.014**	*F*_2,290_ = 9.34 *p* = **.0001**	*F*_2,288_ = 50.9 *p* < **.0001**
Sex		*F*_1,288_ = 3.89 *p* = .049	*F*_1,290_ = 2.74 *p* = .099	*F*_1,288_ = 5.67 *p* = **.018**
Year		*F*_1,288_ = 8.73 *p* = **.0034**	*F*_1,290_ = 0.20 *p* = .653	*F*_1,288_ = 2.37 *p* = .125
Age × Year		*F*_2,288_ = 4.54 *p* = **.012**	‐	‐
*R* ^2^		.744	.076	.270
**(b)**				
Juveniles (July)	151	6.18 ± 0.02^a^	10.2 ± 0.02^a^	20.0 ± 0.03^a^
Subadults	70	5.29 ± 0.03^b^	10.1 ± 0.03^b^	19.6 ± 0.04^b^
Adults	74	5.68 ± 0.03^c^	10.3 ± 0.03^a^	19.6 ± 0.04^b^

^†^ *n* = 293, without two outliers in juveniles.

All measures of skull size differed significantly between the age categories (Table 4b). Skull height decreased significantly by 14.4% from summer juveniles to winter subadults and regrew by 7.4% in the following summer adults. Skull width followed this pattern with a lesser but statistically significant decrease of 1.4% and then an increase of 1.9%. The 2% decrease in skull length from juveniles to subadults was also statistically significant (Table 4b).

### 3.4 Change in the magnitude of Dehnel's phenomenon from 1955 to 1985

We compared the slopes of the regression lines of skull height over time (years) between juveniles versus subadults and subadults versus adults (Figure 4a, see Figure S4 for regression lines and scatterplots). The skull height of July juvenile shrews did not change over the tested period (*F*_1,149_ = 1.34, *p* = .249) but decreased significantly in winter subadults (*F*_1,68_ = 19.52, *p* < .0001; Figure 4a), which was reflected in a significant interaction between age and time (years) (*F*_1,217_ = 8.23, *p* = .0045). This means that the decline in skull height from July to winter was greater with time. The interaction was not significant when the regression lines of August juveniles and subadults were compared (*F*_1,118_ = 0.63, *p* = .430). The skull height of adults did not change with time (*F*_1,72_ = 0.00, *p* = .985); considering the steep decrease in subadults, the regrowth in skull height from subadults to adults increased over time (interaction: *F*_1,140_ = 9.54, *p* = .0024; Figure 4a). Sex, when added to these models was not significant.

**FIGURE 6:**
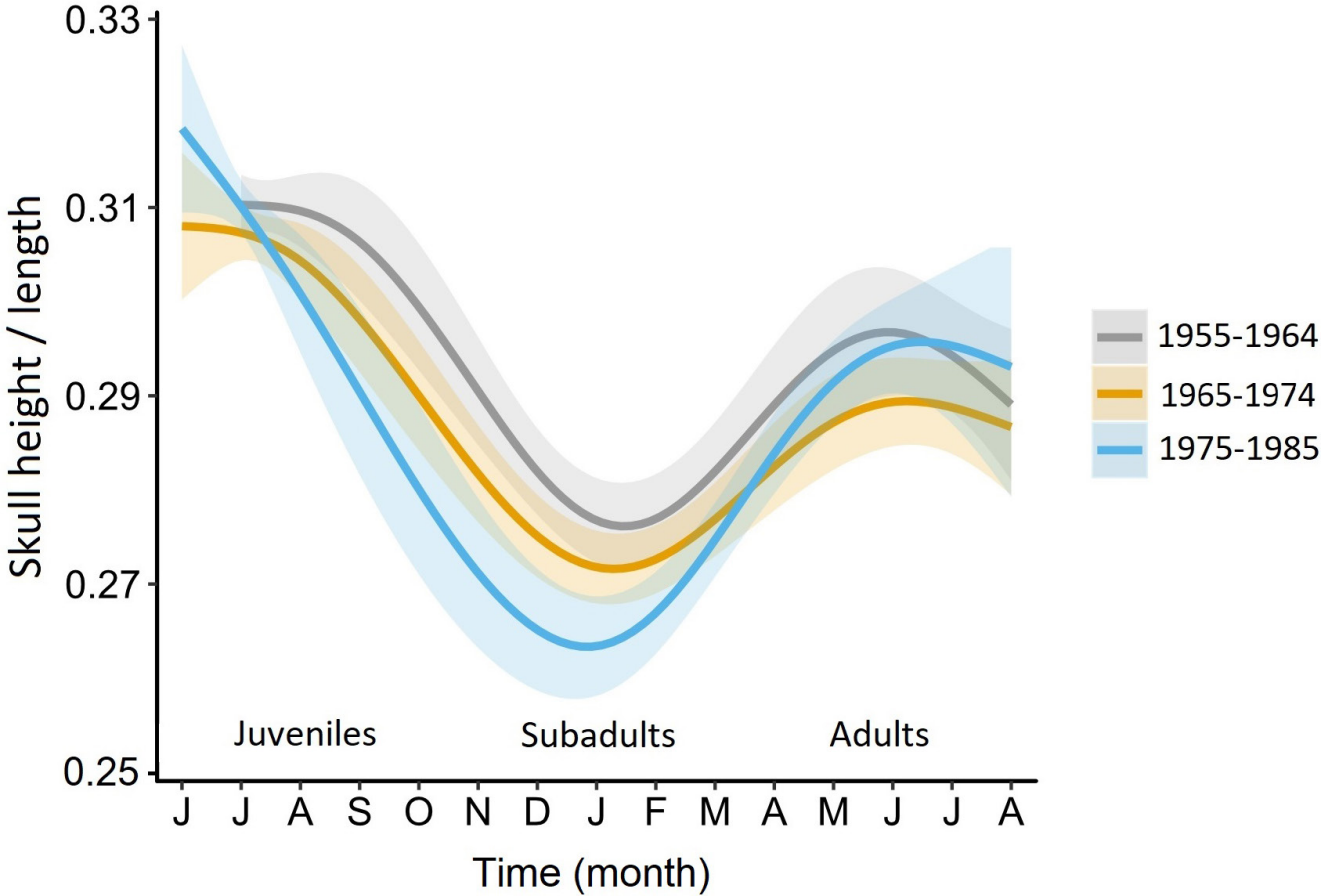
Seasonal change in corrected skull height in *Sorex araneus* over three decades (see Figure 3 for information on the shrews' life history). Solid lines and shaded areas represent fitted values and standard errors of the model (GAM, *n* = 372, e.d.f. (smooth term 1955–1963) = 2.96, e.d.f. (smooth term 1964–1974) = 2.96, e.d.f. (smooth term 1975–1985) = 3.37, *p* < .001 for the three decades, deviance explained 64.9%).

This phenomenon is also illustrated in Figure 6, which depicts the change in shrew skull height corrected for skull length during its lifetime over three decades. The standard errors of juvenile and adult skull height overlapped considerably, while subadult skull height decreased strongly over time (years). In summary, the magnitude of Dehnel's phenomenon increased over the tested period due to winter animals becoming smaller.

## 4 DISCUSSION

### 4.1 Decrease in skull and thus overall size over time (1953–2004)

Skull dimensions, skull length, the often‐used proxy for overall body size, as well as skull height and width, decreased significantly in juvenile and adult *S. araneus* during the study period. We found sexual dimorphism in skull size; males had larger skulls than females, but the sexes did not differ in the degree of decrease in skull size. Our results add to the very mixed picture of the change in body size in mammals under global climate change. The analysis of 50 species of rodents from seven families revealed no significant temporal change in body size (represented mainly by skull dimensions) in 29 species, a significant decline in 13 (26% of the total number), and an increase in size in eight species (16% of the total) (Nengovhela et al., 2020). Of the three carnivore species in which skull size was measured in the second half of the 20th century and analyzed in the context of climate change, skull size showed a significant increase in two species and a significant decline in the other (Yom‐Tov et al., 2008; Yom‐Tov, Leader, et al., 2010; Yom‐Tov, Roos, et al., 2010). Thus, *S. araneus* in Białowieża adds to the number of rather rare cases of decreased body sizes among mammals. However, when body mass is analyzed in terrestrial mammals, a clear tendency of the decrease in body mass is observed; this is also true for shrews in the 20th century (Naya et al., 2017).

The decrease in body size in *S. araneus* in Białowieża is in contrast with the observed increased body size (body and foot length) in *S. cinereus* in Alaska during the second half of the twentieth century along with increasing temperature. In *S. cinereus*, the increase was presumably due to higher food availability in winter as a result of higher winter temperatures, favorable for its prey (Yom‐Tov & Yom‐Tov, 2005), which consists mainly of insect larvae, beetles and spiders (Whitaker, 2004). We argue that the smaller size of *S. araneus* in summer could be an effect of increasing drought leading to reduced availability of earthworms, its main food source. The negative impact of decreased soil moisture on the abundance of epigeic and endogeic invertebrates, especially earthworms, has been well documented (Coyle et al., 2017; Singh et al., 2019). Earthworms have very limited morphological and physiological means for reducing water loss through the cuticle and need moist soil to avoid dehydration (Carley, 1978). *Sorex araneus* strongly depends on a high abundance of food because its metabolic rates measured under natural conditions equal 258% of the value predicted for an average mammal of the same body mass (Ochocińska & Taylor, 2005). Consequently, *S. araneus* prefers humid habitats with humus‐rich soil (Shchipanov et al., 2019), such as our study plot, which harbor large numbers of earthworms.

In support of this, the decrease in skull length in adults between 1970 and 2004 was significantly correlated with the increase in soil moisture deficit (Figure 5), which started to increase in the Białowieża Forest in the 1970s and accelerated in the 1980s as a result of decreasing precipitation and increasing temperatures (Figure 4c–e). The decrease in skull size in both juveniles and adults was stronger after 1970, which coincided with the inflection point in precipitation and soil moisture deficit (Figure 4). Other correlations of skull size with moisture deficit and precipitation in juveniles and adults presented in Table 3 also support the importance of these two climate parameters for the long‐term decrease in body size of *S. araneus*.

There were no regular measurements of the soil water level in the Białowieża Forest before 1985, and those that existed were not comparable with later measurements. However, between 1985 and 2001, the water level decreased in mixed deciduous biotopes by 40 cm. This resulted from an increase in temperature, a decrease in rainfall, and winters without snow and thus a lack of water accumulation at the beginning of the vegetation period (Pierzgalski et al., 2002). The progressive decrease in the soil water level was certainly magnified by the intense drainage work on peatlands in the eastern Belorussian part of the Białowieża Forest in the late 1950s. The average biomass of earthworms in the upper soil layer in the mixed deciduous areas of the Białowieża Forest was 42.2 g m^−2^ between 1997 and 2000 and depended on soil moisture: it was highest in spring, decreased to 22% of the spring values in summer, and increased to 41% in autumn (Kowalczyk et al., 2003). This pattern followed the annual course of soil humidity in the Białowieża mixed deciduous forest (Pierzgalski et al., 2002). Earthworms were not available or were hardly available in the forest in winter (Kowalczyk et al., 2003).

The question arises of whether *S. araneus* can compensate for the low availability of earthworms caused by drought and indicated by its increasingly smaller size. To some extent, *S. araneus* can replace the missing earthworms with other, less preferred prey. Churchfield et al. (2012) demonstrated that *S. araneus* eat diplopods and tiny mites in winter, which are completely ignored in summer, when earthworms are available. In particular, diplopods are unprofitable prey because they secrete a range of defensive toxins and irritants and have a high indigestible chitin content. Low availability of earthworms may also cause a decrease in the population density of *S. araneus* and result in a better fit to food resources. The population density of the badger (*Meles meles*), which depends on earthworms in its diet, is higher in habitats in the Białowieża Forest and elsewhere in Europe, where the biomass of earthworms in the soil is high (Kowalczyk et al., 2003). The shift to prey other than earthworms, the reduction in the population density of *S. araneus*, and the observed decrease in body size (and amount of consumed food) may explain why no change in the breeding phenology of *S. araneus* was detected (see Section 2) despite climate change.

While the increasing soil moisture deficit and decreasing precipitation affected both juveniles and adults, resulting in smaller skull size, the adults were additionally affected by the conditions in the preceding winter. Milder minimum temperatures in February and a decreasing number of days with snow cover in the coldest months of January and February resulted in adults with lower skull heights (Table 3). The lack of snow cover leads to freezing of the upper soil layer and substantially reduces the abundance of various arthropods, which are important components of the winter diet in *S. araneus* (Templer et al., 2012). Snow generates a relatively mild and stable subnivean microclimate in the litter layer where shrews live (Churchfield et al., 2012; Coulianos & Johnels, 1962), which is not only favorable for their prey but may also diminish the costs of thermoregulation and consequently the food requirements of the shrews (Aitchison, 1987).

Skull dimensions in juvenile *S. araneus* from the southern part of the Komi Republic of Russia were correlated with temperature and precipitation from 1976 to 2003, but in contrast to our results, there was no clear directional trend to the change (Poroshin et al., 2010). This might have been caused by the lack of clear change in weather parameters and/or the shorter time period covered in that study than in ours.

Recent evidence suggests a relationship between the tendency of body size to decline over time and metabolic rates in small mammals. In rodents that do not use torpor, there is a significant negative correlation between the temporal body mass change and basal metabolic rate (BMR; Villar & Naya, 2018). Species with a high BMR have decreased in the last six decades. The size decrease in *S. araneus*, with their lack of ability to hibernate, and the BMR in juvenile *S. araneus*, which equals 278% of the value predicted for an average mammal of the same body mass (Ochocińska & Taylor, 2005), fits this pattern. It is likely that the relationship between body size decrease and high metabolic rate is generally true for soricine shrews. Three out of four American *Sorex* species (not including *S. cinereus*) had a decreased body mass during the 20th century, and two of them had a significantly decreased body mass (Naya et al., 2017). The body mass of the short‐tailed shrew (*Blarina brevicauda*) also decreased in 20th century (Naya et al., 2017). However, data from different ages and thus Dehnel stages were pooled in this study, potentially affecting the results and hiding the effects of seasonal body size changes.

### 4.2 Changes in the magnitude of Dehnel's phenomenon (1955–1985)

We found that the decline in skull height from summer to winter in *S. araneus* from the Białowieża Forest became stronger between 1955 and 1985 (Figure 6). We standardized skull height with skull length in the analysis of Dehnel's phenomenon to describe changes in relative skull height. Relative skull height is strongly correlated with brain size (Bielak & Pucek, 1960; Lazáro et al., 2018). Thus, our results also reflected changes in the magnitude of brain size changes. Initially, the skull height of juveniles did not differ between July and August from 1955 to the 1970s. The skull height in these 2 months then diverged over the following years (Figure 4a). The smaller skull height in August than in July after 1970 meant that the decrease in skull height started earlier, already in summer. The magnitude of the decrease was also higher because the relative skull height of winter subadults decreased even more over time (Figure 6).

What is the environmental background of these temporal changes? The increasing magnitude of Dehnel's phenomenon was largely due to the significant decline in the skull height in January and February subadults (Figure 4a). Unfortunately, the numbers of available subadult specimens each year were too low to statistically correlate them with weather conditions. The significant decrease in January temperatures until 1972 might explain the increasing magnitude of Dehnel's phenomenon, but January temperatures did not change significantly later on. A seasonal decrease in skull height in *S. araneus* is thought to be anticipatory of harsh winter conditions. The question then arises whether weather conditions in summer and autumn can have direct or indirect impacts on skull height in winter. Mean daily July temperatures did not change from 1955 to 1985, but August temperatures significantly increased in this period. One may speculate that increasing temperatures and possibly drought could decrease the availability of earthworms and other invertebrates in August and accelerate the decrease in skull height over time (as discussed above), leading to a decrease in skull height in winter. A lower amount of invertebrate food in summer could lead to a greater reduction in resources in winter. Although we did not find a significant change in the soil moisture deficit in August between 1955 and 1985, the sharper skull height decrease with the increasing mean temperature in the driest quarter of the year in a geographic comparison of *S. araneus* (Lazáro et al., 2021) supported the hypothesis that soil moisture deficit in summer might be an important factor for this species. To elucidate this, the role of the availability of food in summer and winter in Dehnel's phenomenon should be tested under controlled laboratory conditions.

We also found a significantly greater skull height regrowth from 1955 to 1985, which resulted from a steep decrease in skull height in the subadults, while adults did not change (Figure 4a). Almost nothing is known about the drivers of this regrowth, which are presumably different from those of the decrease and are likely associated with preparation for the terminal reproductive period in shrews (Lazáro et al., 2019, 2021). Comparison of the skull height regrowth in different *S. araneus* populations revealed only one correlation with weather parameters, namely, a positive correlation with precipitation during the warmest quarter of the year (Lazáro et al., 2021). This also points to changes in food availability.

In summary, we documented a decrease in overall size as represented by absolute skull height and length in the 52‐year (1953–2004) skull series and identified increasing temperature and drought as the main correlates of the decrease. Interpreting the results regarding Dehnel's phenomenon, which increased in magnitude because of a sharp size decrease in winter subadults, was more difficult. Our dataset about winter subadults stopped shortly before the time point when climate change became significant, and it would have been interesting to see how subadult size developed after this. There are strong indications that there are important effects of climate on food availability that indirectly cause trends in both skull size and the magnitude of Dehnel's phenomenon. However, this must be clarified experimentally.

With continued climate change, all the parameters that we found to be of importance for the size of the common shrew and its unique winter adaptation are expected to increase even more strongly. This may negatively affect populations of *S. araneus* in many regions that may become too hot and/or dry for these cold‐adapted animals.

## AUTHOR CONTRIBUTIONS

**Jan R. E. Taylor:** Conceptualization (lead); formal analysis (lead); investigation (lead); project administration (supporting); supervision (supporting); visualization (lead); writing – original draft (lead); writing – review and editing (lead). **Marion Muturi:** Data curation (lead); writing – original draft (supporting); writing – review and editing (supporting). **Javier Lázaro:** Conceptualization (equal); methodology (equal); visualization (supporting); writing – review and editing (supporting). **Karol Zub:** Conceptualization (equal); formal analysis (supporting); resources (supporting); writing – original draft (supporting); writing – review and editing (supporting). **Dina K. N. Dechmann:** Conceptualization (lead); methodology (equal); project administration (lead); supervision (lead); visualization (supporting); writing – original draft (equal); writing – review and editing (supporting).

## CONFLICT OF INTEREST

The authors declare that they have no conflicts of interest.

## Supporting information


Figures S1–S4

## Data Availability

The primary data on skull sizes and climate in Białowieża can be found in Dryad: https://doi.org/10.5061/dryad.xd2547dm7.

## References

[ece39447-bib-0001] Aiello, L. C. , & Wheeler, P. (1995). The expensive‐tissue hypothesis. Current Anthropology, 36, 199–221. 10.1086/204350

[ece39447-bib-0002] Aitchison, C. W. (1987). Winter energy requirements of soricine shrews. Mammal Review, 17, 25–38. 10.1111/j.1365-2907.1987.tb00046.x

[ece39447-bib-0003] Ashton, K. G. (2002). Patterns of within‐species body size variation of birds: Strong evidence for Bergmann's rule. Global Ecology and Biogeography, 11, 505–523. 10.1046/j.1466-822X.2002.00313.x

[ece39447-bib-0004] Ashton, K. G. , Tracy, M. C. , & De Queiroz, A. (2000). Is Bergmann's rule valid for mammals? The American Naturalist, 156, 390–415. 10.1086/303400 29592141

[ece39447-bib-0005] Bielak, T. , & Pucek, Z. (1960). Seasonal changes in the brain weight of the common shrew (*Sorex araneus araneus* Linnaeus, 1758). Acta Theriologica, 3, 297–300.

[ece39447-bib-0006] Boutin, S. , & Lane, J. E. (2014). Climate change and mammals: Evolutionary versus plastic responses. Evolutionary Applications, 7, 29–41. 10.1111/eva.12121 24454546 PMC3894896

[ece39447-bib-0007] Canale, C. I. , Ozgul, A. , Allaine, D. , & Cohas, A. (2016). Differential plasticity of size and mass to environmental change in a hibernating mammal. Global Change Biology, 22, 3286–3303. 10.1111/gcb.13286 26994312

[ece39447-bib-0008] Carley, W. W. (1978). Water economy of the earthworm *Lumbricus terrestris* L.: Coping with the terrestrial environment. Journal of Experimental Zoology, 205, 71–78.

[ece39447-bib-0009] Churchfield, S. (1990). The natural history of shrews (pp. 1–178). Christopher Helm.

[ece39447-bib-0010] Churchfield, S. , Rychlik, L. , & Taylor, J. R. E. (2012). Food resources and foraging habits of the common shrew, *Sorex araneus*: Does winter food shortage explain Dehnel's phenomenon? Oikos, 121, 1593–1601. 10.1111/j.1600-0706.2011.20462.x

[ece39447-bib-0011] Cleveland, W. S. (1979). Robust locally weighted regression and smoothing scatterplots. Journal of the American Statistical Association, 74, 829–836.

[ece39447-bib-0012] Coulianos, C. C. , & Johnels, A. G. (1962). Note on the subnivean environment of small mammals. Arkiv för Zoologi, 15, 363–370.

[ece39447-bib-0013] Coyle, D. R. , Nagendra, U. J. , Taylor, M. K. , Campbell, J. H. , Cunard, C. E. , Joslin, A. H. , Mundepi, A. , Phillips, C. A. , & Callaham, M. A. (2017). Soil fauna responses to natural disturbances, invasive species, and global climate change: Current state of the science and a call to action. Soil Biology & Biochemistry, 110, 116–133. 10.1016/j.soilbio.2017.03.008

[ece39447-bib-0014] Dechmann, D. K. N. , LaPoint, S. , Dullin, C. , Hertel, M. , Taylor, J. R. E. , Zub, K. , & Wikelski, M. (2017). Profound seasonal shrinking and regrowth of the ossified braincase in phylogenetically distant mammals with similar life histories. Scientific Reports, 7, 42443. 10.1038/srep42443 28211896 PMC5304206

[ece39447-bib-0015] Dehnel, A. (1949). Studies on the genus *Sorex* L. Annales Universitatis Mariae Curie‐Skłodowska, Sectio C, 4, 18–102. 24538596

[ece39447-bib-0016] Gardner, J. L. , Peters, A. , Kearney, M. R. , Joseph, L. , & Heinsohn, R. (2011). Declining body size: A third universal response to warming? Trends in Ecology & Evolution, 26, 285–291. 10.1016/j.tree.2011.03.005 21470708

[ece39447-bib-0017] Hanski, I. (1994). Population biological consequences of body size in *Sorex* . In J. F. Merritt , G. L. Kirkland, Jr. , & R. K. Rose (Eds.), Advances in the biology of shrews (Vol. 18, pp. 15–26). Carnegie Museum Natural History, Special Publication.

[ece39447-bib-0018] Hume, I. D. , Beiglböck, C. , Ruf, T. , Frey‐Roos, F. , Bruns, U. , & Arnold, W. (2002). Seasonal changes in morphology and function of the gastrointestinal tract of free‐living alpine marmots (*Marmota marmota*). Journal of Comparative Physiology B, 172, 197–207. 10.1007/s00360-001-0240-1 11919701

[ece39447-bib-0019] Hyvärinen, H. (1984). Wintering strategy of voles and shrews in Finland. In J. F. Merritt (Ed.), Winter ecology of small mammals (pp. 139–148). Carnegie Museum of Natural History, Special Publication 10.

[ece39447-bib-0020] Isler, K. , & van Schaik, C. P. (2006). Metabolic costs of brain size evolution. Biology Letters, 2, 557–560. 10.1098/rsbl.2006.0538 17148287 PMC1834002

[ece39447-bib-0021] Keicher, L. , O'Mara, M. T. , Voigt, C. C. , & Dechmann, D. K. N. (2017). Stable carbon isotopes in breath reveal fast metabolic incorporation rates and seasonally variable but rapid fat turnover in the common shrew (*Sorex araneus*). Journal of Experimental Biology, 220, 2834–2841. 10.1242/jeb.159947 28546508

[ece39447-bib-0022] Kowalczyk, R. , Zalewski, A. , Jędrzejewsk, A. B. , & Jędrzejewski, W. (2003). Spatial organization and demography of badgers (*Meles meles*) in Białowieża Primeval Forest, Poland, and the influence of earthworms on badger densities in Europe. Canadian Journal of Zoology, 81, 74–87. 10.1139/z02-233

[ece39447-bib-0061] LaPoint, S. , Keicher, L. , Wikelski, M. , Zub, K. , & Dechmann, D. K. N. (2017). Growth overshoot and seasonal size changes in the skulls of two weasel species. Royal Society Open Science, 4, 160947. 10.1098/rsos.160947 28280592 PMC5319358

[ece39447-bib-0023] Lazáro, J. , Dechmann, D. K. N. , LaPoint, S. , Wikelski, M. , & Hertel, M. (2017). Profound reversible seasonal changes of individual skull size in a mammal. Current Biology, 27, R1089–R1107. 10.1016/j.cub.2017.08.055 29065289

[ece39447-bib-0024] Lazáro, J. , Hertel, M. , LaPoint, S. , Wikelski, M. , Stiehler, M. , & Dechmann, D. K. N. (2018). Cognitive skills of common shrews (*Sorex araneus*) vary with seasonal changes in skull size and brain mass. Journal of Experimental Biology, 221, jeb166595. 10.1242/jeb.166595 29170257

[ece39447-bib-0025] Lazáro, J. , Hertel, M. , Muturi, M. , & Dechmann, D. K. N. (2019). Seasonal reversible size changes in the braincase and mass of common shrews are flexibly modified by environmental conditions. Scientific Reports, 9, 2489. 10.1038/s41598-019-38884-1 30792434 PMC6385354

[ece39447-bib-0026] Lazáro, J. , Nováková, L. , Hertel, M. , Taylor, J. R. E. , Muturi, M. , Zub, K. , & Dechmann, D. K. N. (2021). Geographic patterns in seasonal changes of body mass, skull and brain size of common shrews. Ecology and Evolution, 11, 2431–2448. 10.1002/ece3.7238 33767812 PMC7981214

[ece39447-bib-0027] McNab, B. K. (1991). The energy expenditure of shrews. In J. S. Findley & T. L. Yates (Eds.), The biology of the Soricidae (pp. 35–45). The Museum of Southwestern Biology, Special Publication 1.

[ece39447-bib-0028] McNab, B. K. (2010). Geographic and temporal correlations of mammalian size reconsidered: A resource rule. Oecologia, 164, 13–23. 10.1007/s00442-010-1621-5 20364270

[ece39447-bib-0029] Mezhzherin, V. A. (1964). Dehnel's phenomenon and its possible explanation. Acta Theriologica, 8, 95–114 (in Russian with English summary).

[ece39447-bib-0030] Naya, D. E. , Naya, H. , & Cook, J. (2017). Climate change and body size trends in aquatic and terrestrial endotherms: Does habitat matter? PLoS One, 12, e0183051. 10.1371/journal.pone.0183051 28813491 PMC5558942

[ece39447-bib-0031] Nengovhela, A. , Denys, C. , & Taylor, P. J. (2020). Life history and habitat do not mediate temporal changes in body size due to climate warming in rodents. PeerJ, 8, e9792. 10.7717/peerj.9792 33024624 PMC7520088

[ece39447-bib-0032] Ochocińska, D. , & Taylor, J. R. E. (2003). Bergmann's rule in shrews: Geographical variation of body size in Palearctic *Sorex* species. Biological Journal of the Linnean Society, 78, 365–381. 10.1046/j.1095-8312.2003.00150.x

[ece39447-bib-0033] Ochocińska, D. , & Taylor, J. R. E. (2005). Living at the physiological limits: Field and maximum metabolic rates of the common shrew (*Sorex araneus*). Physiological and Biochemical Zoology, 78, 808–818. 10.1086/431190 16096983

[ece39447-bib-0034] Pankakoski, E. (1989). Variation in the tooth wear of the shrews *Sorex araneus* and *S. minutus* . Annales Zoologici Fennici, 26, 445–457.

[ece39447-bib-0035] Parmesan, C. (2006). Ecological and evolutionary responses to recent climate change. Annual Review of Ecology, Evolution, and Systematics, 37, 637–669. 10.1146/annurev.ecolsys.37.091305.110100

[ece39447-bib-0036] Piersma, T. , Gudmundsson, G. , & Lilliendahl, K. (1999). Rapid changes in the size of different functional organ and muscle groups during refueling in a long‐distance migrating shorebird. Physiological and Biochemical Zoology, 72, 405–415. 10.1086/316680 10438678

[ece39447-bib-0037] Pierzgalski, E. , Boczoń, A. , & Tyszka, J. (2002). Variability of precipitation and ground water level in the Białowieża National Park. Kosmos, 51, 415–425 (in Polish with English summary).

[ece39447-bib-0038] Poroshin, E. A. , Polly, P. D. , & Wójcik, J. M. (2010). Climate and morphological change on decadal scales: Multiannual variation in the common shrew *Sorex araneus* in Northeastern Russia. Acta Theriologica, 55, 193–202. 10.4098/j.at.0001-7051.106.2009

[ece39447-bib-0039] Pucek, Z. (1963). Seasonal changes in the braincase of some representatives of the genus *Sorex* from the Palearctic. Journal of Mammalogy, 44, 523–536. 10.2307/1377135

[ece39447-bib-0040] Pucek, Z. (1965). Seasonal and age changes in the weight of internal organs in shrews. Acta Theriologica, 10, 369–438.

[ece39447-bib-0041] Pucek, Z. (1970). Seasonal and age change in shrews as an adaptive process. Symposia of the Zoological Society of London, 26, 189–207.

[ece39447-bib-0042] Pucek, Z. (1981). Order insectivores – Insectivora. In Z. Pucek (Ed.), Keys to vertebrates of Poland: Mammals (pp. 62–101). PWN – Polish Scientific Publishers.

[ece39447-bib-0043] R Core Team . (2016). R: A language and environment for statistical computing, version 3.3.1. R Foundation for Statistical Computing.

[ece39447-bib-0044] Sauer, J. R. , & Slade, N. A. (1987). Size‐based demography of vertebrates. Annual Review of Ecology and Systematics, 18, 71–90. 10.1146/annurev.es.18.110187.000443

[ece39447-bib-0045] Schaeffer, P. J. , O'Mara, M. T. , Breiholz, J. , Keicher, L. , Lázaro, J. , Muturi, M. , & Dechmann, D. K. N. (2020). Metabolic rate in common shrews is unaffected by temperature, leading to lower energetic costs through seasonal size reduction. Royal Society Open Science, 7, 191989. 10.1098/rsos.191989 32431881 PMC7211839

[ece39447-bib-0046] Shchipanov, N. A. , Zima, J. , & Churchfield, S. (2019). Introducing the common shrew. In J. B. Searle , P. D. Polly , & J. Zima (Eds.), Shrews, chromosomes and speciation (pp. 19–67). Cambridge University Press.

[ece39447-bib-0047] Singh, J. , Schädler, M. , Demetrio, W. , Brown, G. G. , & Eisenhauer, N. (2019). Climate change effects on earthworms ‐ A review. Soil Organisms, 91, 113–137. 10.25674/so91iss3pp114 PMC6944501 31908681

[ece39447-bib-0048] Taylor, J. R. E. (1998). Evolution of energetic strategies in shrews. In J. M. Wójcik & M. Wolsan (Eds.), Evolution of shrews (pp. 309–346). Mammal Research Institute Polish Academy of Sciences.

[ece39447-bib-0049] Taylor, J. R. E. , Rychlik, L. , & Churchfield, S. (2013). Winter reduction in body mass in a very small, nonhibernating mammal: Consequences for heat loss and metabolic rates. Physiological and Biochemical Zoology, 86, 9–18. 10.1086/668484 23303317

[ece39447-bib-0050] Templer, P. H. , Schiller, A. F. , Fuller, N. W. , Socci, A. M. , Campbell, J. L. , Drake, J. E. , & Kunz, T. H. (2012). Impact of a reduced winter snowpack on litter arthropod abundance and diversity in a northern hardwood forest ecosystem. Biology and Fertility of Soils, 48, 413–424. 10.1007/s00374-011-0636-3

[ece39447-bib-0051] Teplitsky, C. , & Millien, V. (2014). Climate warming and Bergmann's rule through time: Is there any evidence? Evolutionary Applications, 7, 156–168. 10.1111/eva.12129 24454554 PMC3894904

[ece39447-bib-0052] Villar, C. H. , & Naya, D. E. (2018). Climate change and temporal trends in body size: The case of rodents. Oikos, 127, 1186–1194. 10.1111/oik.04884

[ece39447-bib-0053] Walther, G. R. , Post, E. , Convey, P. , Menzel, A. , Parmesan, C. , Beebee, T. J. C. , Fromentin, J. M. , Hoegh‐Guldberg, O. , & Bairlein, F. (2002). Ecological responses to recent climate change. Nature, 416, 389–395. 10.1038/416389a 11919621

[ece39447-bib-0054] Whitaker, J. O., Jr. (2004). Sorex cinereus . Mammalian Species, 743, 1–9.

[ece39447-bib-0055] Willmott, C. J. (1977). WATBUG: A FORTRAN IV algorithm for calculating the climatic water budget (Vol. 30, pp. 1–55). CW Thornthwaite Associates Laboratory of Climatology, Publications in Climatology. http://udspace.udel.edu/handle/19716/20487

[ece39447-bib-0056] Yom‐Tov, Y. , & Geffen, E. (2011). Recent spatial and temporal changes in body size of terrestrial vertebrates: Probable causes and pitfalls. Biological Reviews, 86, 531–541. 10.1111/j.1469-185X.2010.00168.x 21070587

[ece39447-bib-0057] Yom‐Tov, Y. , Leader, N. , Yom‐Tov, S. , & Baagøe, H. J. (2010). Temperature trends and recent decline in body size of the stone marten *Martes foina* in Denmark. Mammalian Biology, 75, 146–150. 10.1016/j.mambio.2008.10.005

[ece39447-bib-0058] Yom‐Tov, Y. , Roos, A. , Mortensen, P. , Wiig, Ø. , Yom‐Tov, S. , & Heggberget, T. M. (2010). Recent changes in body size of the Eurasian otter *Lutra lutra* in Sweden. Ambio, 39, 496–503. 10.1007/s13280-010-0074-8 21090004 PMC3357672

[ece39447-bib-0059] Yom‐Tov, Y. , & Yom‐Tov, J. (2005). Global warming, Bergmann's rule and body size in the masked shrew *Sorex cinereus* Kerr in Alaska. Journal of Animal Ecology, 74, 803–808. 10.1111/j.1365-2656.2005.00976.x

[ece39447-bib-0060] Yom‐Tov, Y. , Yom‐Tov, S. , & Jarrell, G. (2008). Recent increase in body size of the American marten *Martes americana* in Alaska. Biological Journal of the Linnean Society, 93, 701–707. 10.1111/j.1095-8312.2007.00950.x

