# Supplementary material for: Fifty years of data show the effects of climate on overall skull size and the extent of seasonal reversible skull size changes (Dehnel's phenomenon) in the common shrew
Source: Ecol Evol. 2022 Oct 27;12(10):e9447. doi: 10.1002/ece3.9447 (PMC9609440; doi:10.1002/ece3.9447)
Supplement: Supplementary file 1 — Figures S1–S4 [file ECE3-12-e9447-s001.docx]

**Electronic Supplemental Material for:**

Fifty years of data show the effects of climate on overall skull size and the extent of seasonal reversible skull size changes (Dehnel’s phenomenon) in the common shrew

Jan R.E. Taylor^*^, Marion Muturi, Javier Lázaro, Karol Zub, Dina K.N. Dechmann

^*^Corresponding author: e-mail: [taylor@uwb.edu.pl](mailto:taylor@uwb.edu.pl)

**Figure S1** The size of skull in females and males of juvenile and adult *Sorex araneus* from Białowieża Forest between 1953 and 2004; a skull height, **b** skull width, **c** skull length

| (a) | 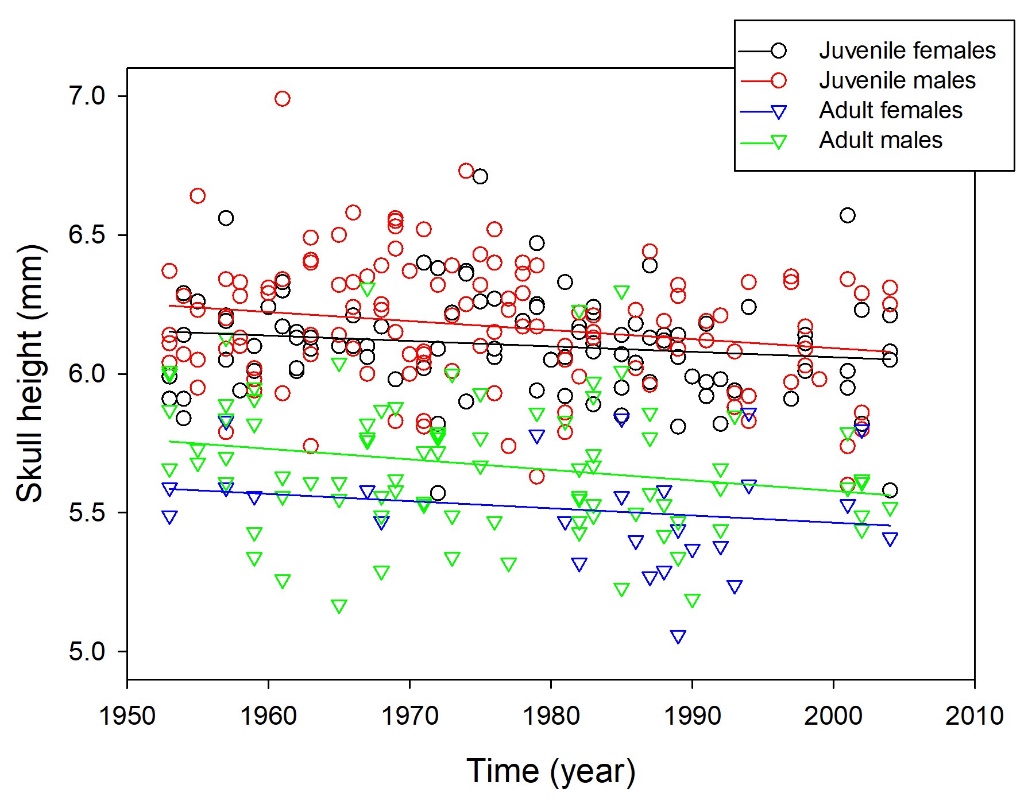 |
| --- | --- |

| (b) | 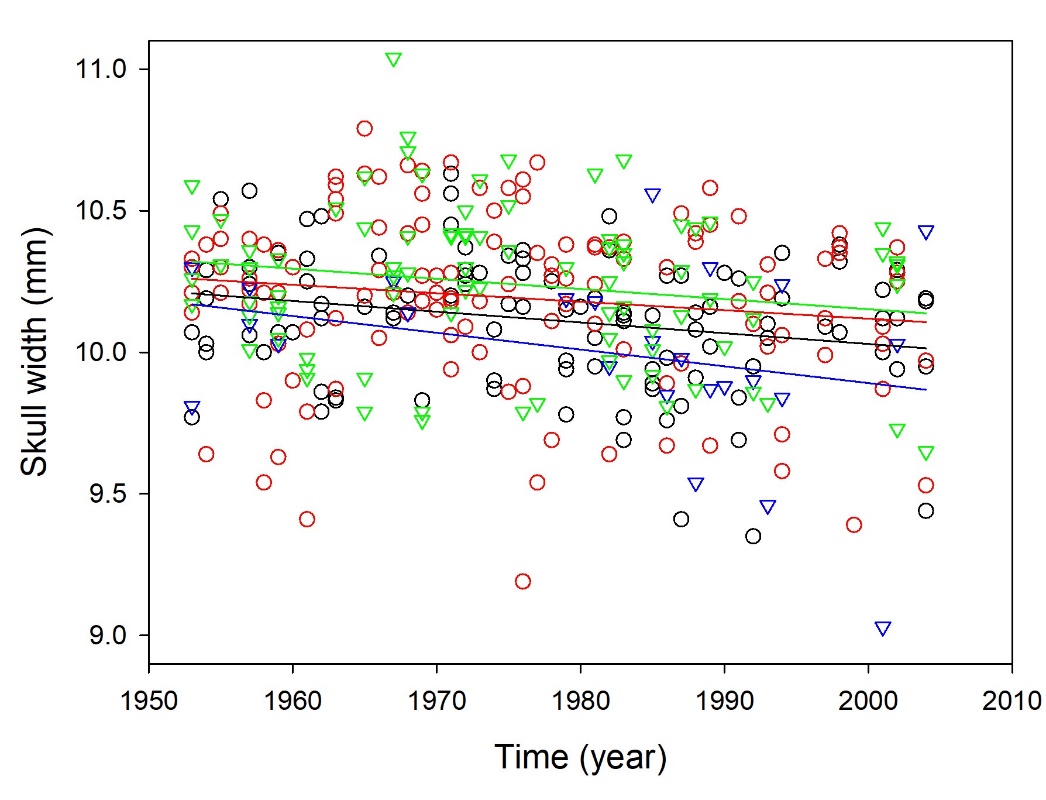 |
| --- | --- |

| (c) | 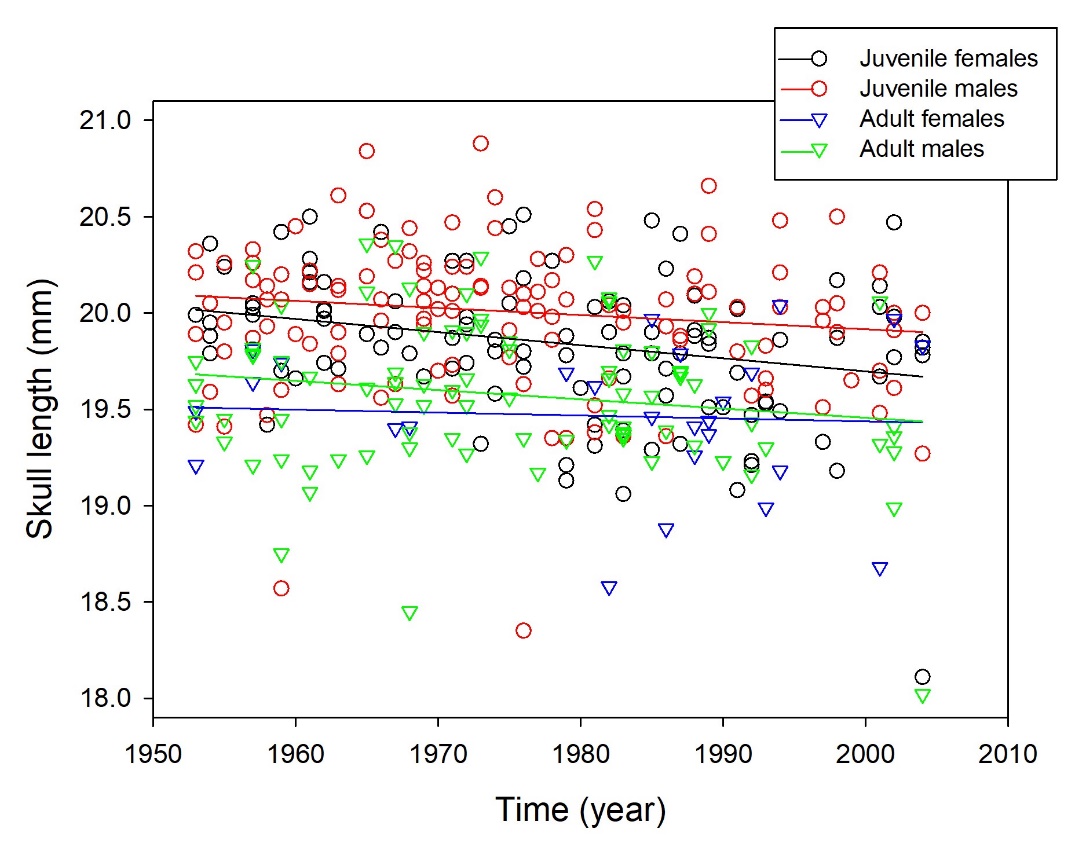 |
| --- | --- |

**Figure S2** The relationships between skull size measurements and time (year) in July juvenile (left panel) and adult (right panel) *S. araneus* from 1953 to 2004. **a**, **b** Skull height, **c**, **d** skull width, **e**, **f** skull length. Confidence limits of relationships smoothed with LOESS are shown

| (a) |  | (b) |
| --- | --- | --- |
| 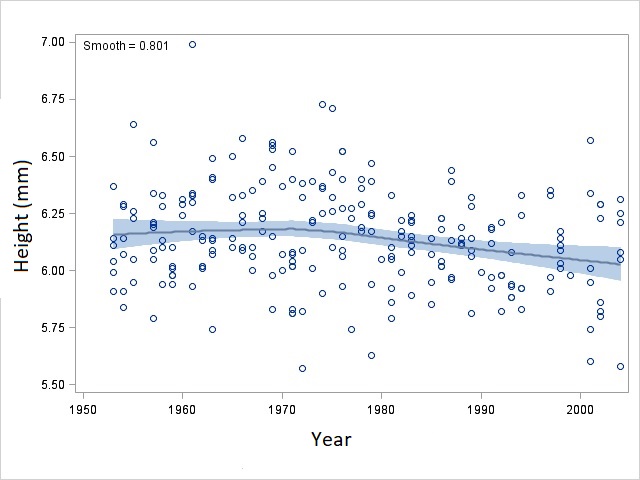 |  | 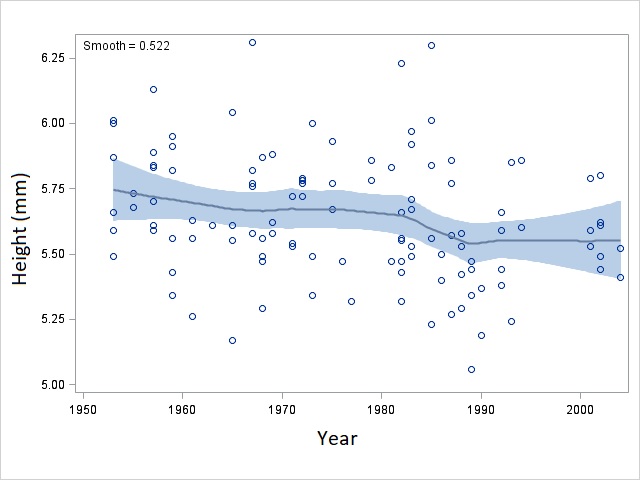 |
| (c) |  | (d) |
| 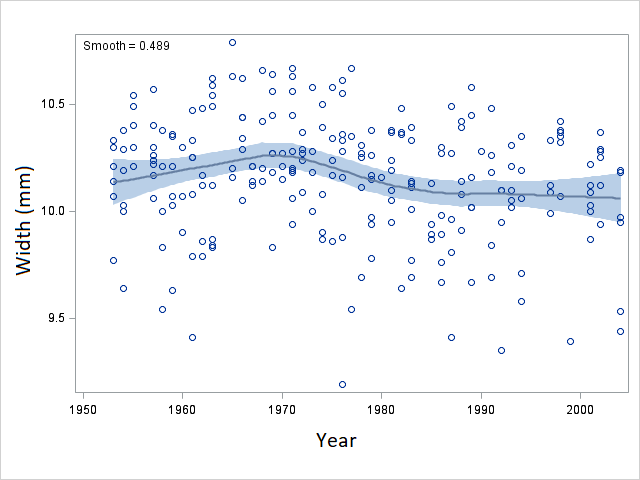 |  | 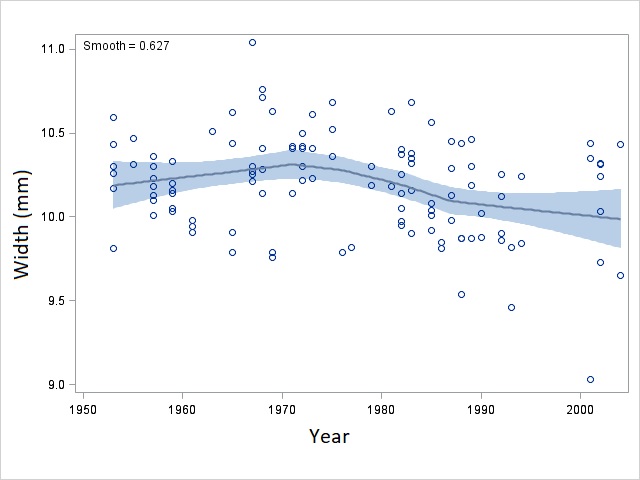 |
| (e) |  | (f) |
| 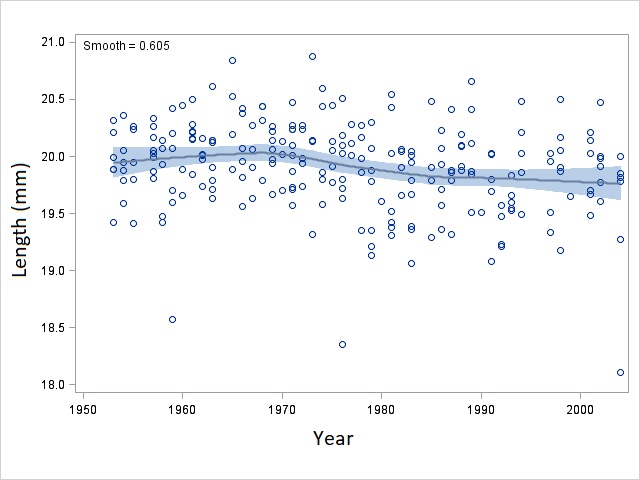 |  | 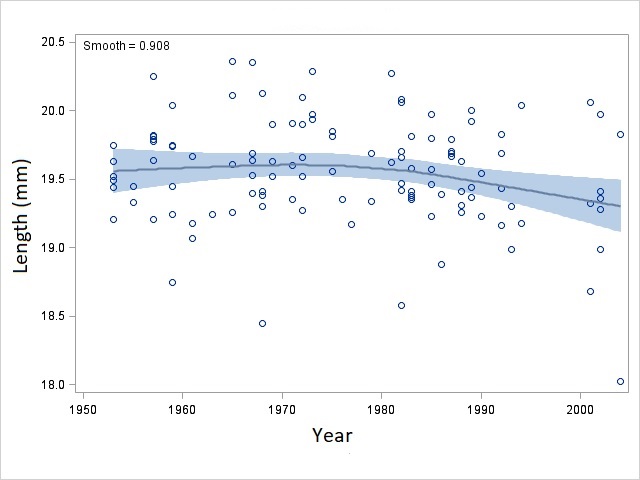 |

| **Figure S3** Trends in weather parameters in Białowieża between 1952 and 2004; **a** mean annual temperature, **b** mean July temperature, **c** mean January temperature, **d** annual sum of precipitation, **e** annual sum of soil moisture deficit, **f** number of days with snow cover. The 95% confidence limits of relationships smoothed with LOESS are shown as shaded areas. 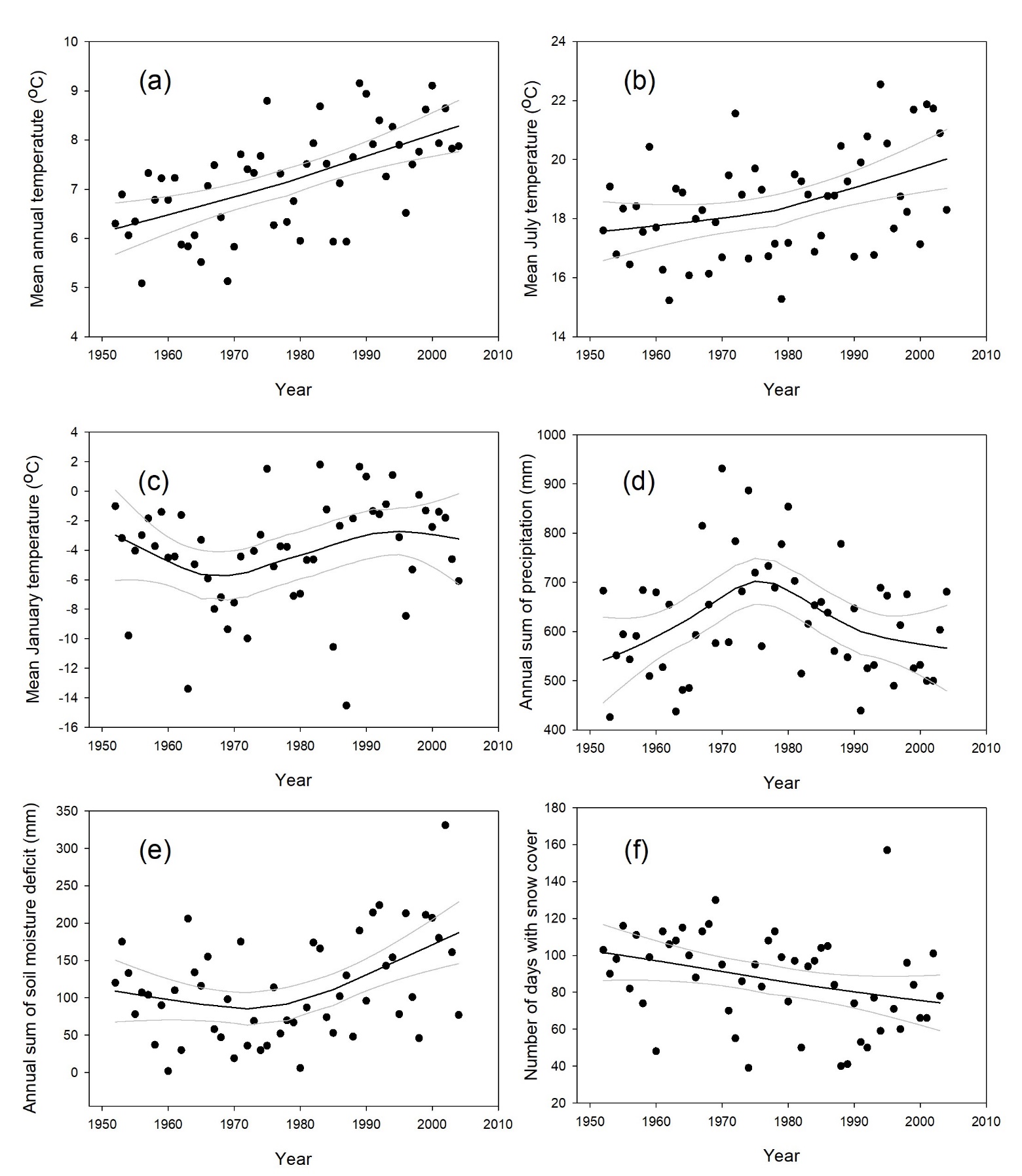 |
| --- |

**Figure S4** Dehnel’s phenomenon in *S. araneus* from 1955 to 1985; **a** differences in skull height between July juveniles (JUV-7, blue) and winter subadults (SAD, red), **b** between August juveniles (JUV-8, blue) and subadults (red), **c** between subadults (blue) and adults (AD, red)

| (a) | 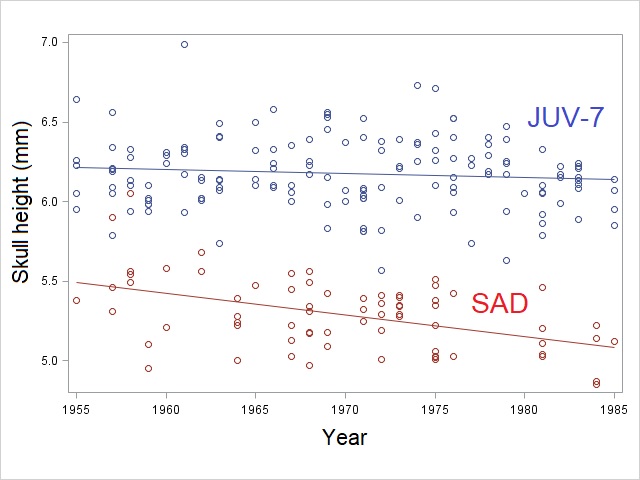 |
| --- | --- |
| (b) | 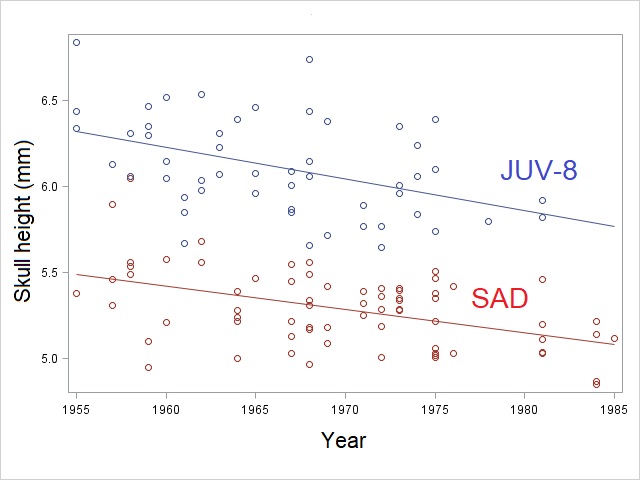 |
| (c) | 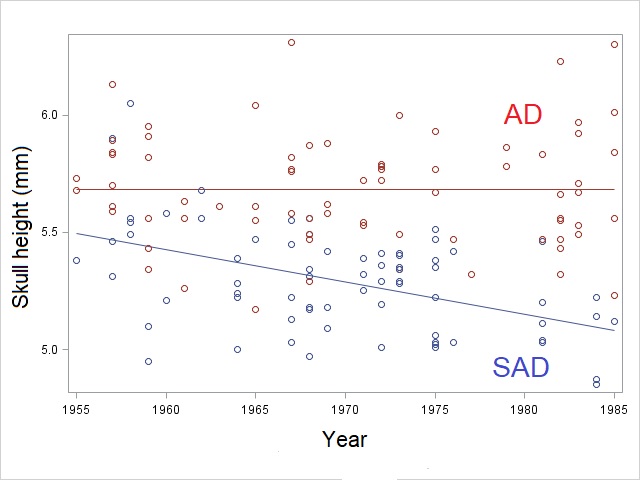 |
